# Association of heart rate variability, exercise intensity and exercising arrhythmias with competition results in eventing horses

**DOI:** 10.1111/evj.14491

**Published:** 2025-02-26

**Authors:** Cristobal Navas de Solis, Alessandra Ramseyer, Darko Stefanovski, Joanne Haughan, Claire J. Solomon, Katharina Kirsch

**Affiliations:** ^1^ Large Animal Clinical Sciences, School of Veterinary Medicine University of Pennsylvania Philadelphia Pennsylvania USA; ^2^ Swiss Institute of Equine Medicine University of Bern Avenches Switzerland; ^3^ German Olympic Committee for Equestrian Sports (DOKR) Warendorf Germany

**Keywords:** detrended fluctuation analysis, electrocardiogram, equestrian sports, horse

## Abstract

**Background:**

Exercising arrhythmias are common in horses participating in eventing competitions. Heart rate variability (HRV) and a specific measure of the degree of heart rate fluctuation (Detrended Fluctuation Analysis alpha1; DFA‐α1) are proposed as exercise intensity and fatigue markers.

**Objectives:**

(1) To describe exercising arrhythmias and DFA‐α1 values during 2–4* level eventing cross‐country competitions in horses from two European countries; (2) to identify associations between exercising arrhythmias, DFA‐α1 and competition results and (3) to evaluate whether markers of the intensity of exercise are associated with exercising arrhythmias, DFA‐α1 or competition results.

**Methods:**

A convenience sample of exercising ECGs and competition results from horses during cross‐country eventing competitions was examined. Statistical analysis was conducted using multivariable mixed‐effects logistic, Poisson and linear regression models.

**Results:**

Arrhythmias were frequent during 69 cross‐country competition ECGs from 43 horses. Detrended fluctuation analysis α1 was not associated with performance during cross‐country, but a higher DFA‐α1 during cross‐country was associated with fewer showjumping phase penalties. More premature complexes, the presence of complex arrhythmias and arrhythmias during recovery were associated with fewer time penalties during cross‐country. The presence of complex arrhythmias and arrhythmias during recovery of the cross‐country phase was associated with more penalties during the showjumping phase. Higher blood lactate concentration, higher HR_peak_ and higher HR_mean_ were associated with fewer time penalties during cross‐country.

**Main Limitations:**

The arrhythmias observed do not allow conclusions to be drawn about the consequences of more severe arrhythmias. The associations described here were often of small magnitude or with large confidence intervals and must be interpreted cautiously.

**Conclusions:**

Arrhythmias during the cross‐country test of eventing competitions were frequently associated with positive competition results during the cross‐country phase and negative results during the showjumping phase. The specific arrhythmia types and circumstances that should raise concern about performance and safety remain relevant but unanswered questions for equine practitioners.

## 1 INTRODUCTION

Exercising arrhythmias are common in horses performing to expectation and not showing signs of disease.^1^, ^2^, ^3^, ^4^, ^5^, ^6^, ^7^ In a recent report, the age, level of the horse and intensity of exercise, estimated by the heart rate, were predictive of various arrhythmia groupings.^7^ Arrhythmias have the potential to suddenly decrease cardiac output and, intuitively, this can decrease muscle and brain perfusion, affecting performance and safety.^8^ Furthermore, it is proposed, yet not proven, that cardiac disease is a leading cause of sudden death and that arrhythmogenesis may be a common reason for sudden cardiac death.^5^, ^9^ The incidence of death during exercise in eventing FEI sanctioned events is 0.58/1000 starts.^10^ Excluding musculoskeletal disease, the incidence of death (often called sudden death) is 0.19/1000 starts.^10^ A better understanding of the clinical and performance relevance of exercising arrhythmias would help clinicians and riders evaluate poor performance and assess safety in an already intrinsically dangerous sport like eventing.

Heart rate variability (HRV) during exercise and, specifically, Detrended Fluctuation Analysis alpha1 (DFA‐α1) has been proposed as markers of exercise intensity and fatigue and shown to be associated with traditional variables, like blood lactate concentration, maximal lactate steady state, or lactate and ventilatory thresholds, used for exercise testing in horses and humans.^11^, ^12^, ^13^ Mathematically, DFA‐α1 describes the root mean square fluctuation of integrated and detrended data from multiple sizes of observation windows. Data are plotted against window size using a log–log scale, and *α* − 1 illustrates the slope of the line describing the relationship between (log) fluctuation and (log) window size.^14^ Mechanistically, the changes in DFA‐α1, like other HRV variables, are proposed to be a consequence of the balance between the arms of the autonomic nervous system. As exercise intensity increases, the sympathetic branch is enhanced, and the parasympathetic branch is withdrawn.^14^, ^15^, ^16^, ^17^ With the initiation of low‐intensity exercise, DFA‐α1 increases and then decreases as exercise becomes more intense. It has been suggested that this progression reflects a protective feedback mechanism signalling that the coordination of subsystems involved in the exercising athlete is failing before the whole system fails.^18^, ^19^

Detrended fluctuation analysis α1 has been shown to be associated with thresholds that mark the transition from moderate to heavy and from heavy to severe exercise intensity in humans.^18^, ^19^, ^20^ Different thresholds such as the aerobic and anaerobic thresholds, thresholds associated with the speed at lactate concentrations of 2 mmol/L (VLa2) or 4 mmol/L (VLa4),^21^, ^22^ maximum lactate steady state^23^ and lactate minimal speed^24^ have been proposed to mark transitions between exercise intensities in humans^25^ and horses.^26^, ^27^ Nomenclature, physiological mechanisms as well as the most appropriate tests to determine these thresholds and exercise intensities have been challenged in both humans^28^, ^29^ and horses.^24^ Despite methodological and semantic controversies, these thresholds have been shown to be associated with readiness to perform, predisposition to injury and running ability in horses.^30^, ^31^ Therefore, user‐friendly methods to describe and monitor exercise intensity could impact equine welfare and performance.

The objectives of this study were: (1) to describe exercising arrhythmias and DFA‐α1 values in eventing horses during cross‐country competition; (2) to evaluate whether exercising arrhythmias and DFA‐α1 are associated with competition results and (3) to evaluate whether markers of exercise intensity (blood lactate concentration, heart rate, speed and distance) are associated with exercising arrhythmias, DFA‐α1 and competition results. We hypothesised that: (1) a higher DFA‐α1, suggestive of relatively lower exercise intensity or superior readiness to compete, would be associated with better competition results; (2) complex arrhythmias would be associated with worse competition results and (3) higher exercise intensity during cross‐country competition would be associated with more arrhythmias and a lower DFA‐α1.

## 2 MATERIALS AND METHODS

### 2.1 Study population

A convenience sample of 111 exercising ECGs from 54 horses obtained during cross‐country eventing competition and corresponding competition results for each horse was used in this study. Inclusion criteria were being monitored by the National German Eventing team or participating in a COVID‐linked programme by the Swiss Equestrian Federation for the National Eventing team and riders. ECGs were excluded if the quality of the ECG did not allow evaluation of QRS morphology and determination of RR intervals in 95% or more of the exercising recording. The institutional animal care committee approved all procedures (protocol VD3585) before data collection. Age, sex and percentage (%) of Thoroughbred blood were collected from horses' records.

### 2.2 Equipment

ECGs were recorded using a fitness tracker device (Equimetre™, Arioneo). The device is placed in a pocket on a custom saddle pad and connected to a surcingle with embedded electrodes that sit under the horses' tack. The fitness tracker records a one lead ECG. Heart rate and rhythm results obtained with this device have been validated previously.^32^, ^33^ Data from GPS with defined technical specifications is used to calculate speed and distance.^34^ Independent testing of the specific GPS model and its derived calculations is unavailable.

### 2.3 Electrocardiographic and HRV analysis

ECG recordings during cross‐country competition and during the fast heart rate deceleration period were used for this study. ECGs and R–R intervals were evaluated, manually and using callipers, by one author (CN) after automatic R markings in HRV software (Kubios version 3.5 for Windows; Kubios Oy) were corrected manually by one of two authors (CS and CN). R–R change of 5% was chosen to determine premature complexes based on exceeding normal variation or heart rates exceeding 100 beats/min^35^ expected during cross‐country competition.^36^ The beat correction setting was set at custom, and 5% of RR at peak heart rate for each recording was calculated. As this was 0.013–0.017 s, and the HRV software rounds the custom setting to the closest first decimal point, electronic callipers (https://stanford.edu/~rogersaj/) were used to determine if QRS complexes eliminated at 0.01 s but not at 0.02 s met the 5% prematurity criteria. Pre‐processing HRV settings were set to the default values, including the R–R detrending method, which was kept at “smoothness priors” (Lambda = 500). Noise detection/correction was not used. Detrended fluctuation analysis α1 window width was set to 4 ≤ *n* ≤ 16 beats^37^ window duration was set to 60 s, and a 1 s grid interval was chosen for the duration of the moving window as previously described.^38^ One rolling averaged DFA‐α1 for the cross‐country competition period was used for analysis.

Arrhythmia description followed criteria recently reported in cross‐country competition.^7^ Briefly, the presence of any type and number of premature complexes was defined as ‘ARRHYTHMIA’. The presence of complex arrhythmia was defined as the presence of multiform premature complexes, triplets, salvos, or runs of premature complexes (ARRHYTHMIA_COMPLEX_). The presence of arrhythmia during the competition period was defined as ‘ARRHYTHMIA_EXERCISE_’ and the presence of arrhythmia during the immediate recovery period as ‘ARRHYTHMIA_RECOVERY_’. These categories were analysed as dichotomous variables, and the number of premature complexes (nPC) was analysed as a continuous variable. Premature complexes were described as wide or narrow based on subjective comparison with the appearance of the preceding QRSs. Due to the ECG system only recording one lead and because P waves were inconsistently visible at high heart rates, PCs were not further classified as ventricular or supraventricular. Single PCs with or without a compensatory pause were also described. The pause category (compensatory or non‐compensatory) was ‘reader‐determined’ and obtained using manual and digital callipers on the ECG trace and the R–R tachogram to determine whether the coupling interval + return cycle length was equal or nearly equal to the sum of the previous 2 R–R intervals as previously described.^7^

### 2.4 Competition results and assessment of exercise intensity

The competition results used to describe performance were the total number of penalties after all three phases of competition (total penalties), time penalties acquired during cross‐country (cross‐country time penalties) and penalties acquired during stadium jumping (showjumping penalties). All competition results were analysed as continuous variables, and a lower value described a better result. Variables used as markers of exercise intensity were mean heart rate during the cross‐country phase (HR_mean_); peak heart rate during the cross‐country phase, calculated as the average of the fastest five consecutive instantaneous heart rates (HR_peak_); average speed during the cross‐country phase (unit = 100 m/min); distance covered during the cross‐country phase (unit = 100 m) and blood lactate concentration. Lactate concentration (mmol/L) was measured in venous blood samples collected 10 min after completion of the cross‐country test. Blood samples were collected by venipuncture of the jugular vein into 3 mL plastic syringes. Blood lactate concentration was determined immediately after blood sample collection from whole blood by means of previously reported and validated hand‐held tools (Lactate Photometer Plus DP 110, Diaglobal^39^ in FEI horses and Lactate Pro2, Axon Lab AG Non‐FEI horses).

### 2.5 Data analysis

All analyses were conducted with statistical software (Stata 17MP, StataCorp) using two‐sided tests of hypotheses and a *p*‐value ≤0.05 as the criterion for statistical significance. Tests of normal distribution (Shapiro–Wilk) were performed to determine the extent of skewness of variables. Descriptive analyses included the computation of means and standard deviations (normally distributed data) or medians, interquartile ranges (IQR) and ranges (non‐normally distributed data) of continuous variables. Categorical variables were tabulated and summarised using frequency counts. The sample size was determined by convenience sampling.

To meet the second objective, statistical analysis was conducted using a multivariable mixed‐effects Poisson regression model to assess the association between the fixed effects nPCs, ARRHYTHMIA_EXERCISE_, ARRHYTHMIA_RECOVERY_ and ARRHYTHMIA_COMPLEX_ with outcomes being competition results (dependent variables). To meet the third objective, competition results and arrhythmia categories were set as dependent variables, while markers of exercise intensity of exercise were set as fixed effects. Multivariable mixed‐effects logistic regression models were used when ARRHYTHMIA_EXERCISE_, ARRHYTHMIA_RECOVERY_ and ARRHYTHMIA_COMPLEX_ were the outcomes. Multivariable mixed‐effects Poisson regression models were used when competition results and nPCs were the outcomes, and linear regression was used when DFA‐α1 was the outcome. For all analyses, the individual horse was introduced as a random effect, and age, sex and the percentage (%) of Thoroughbred blood were introduced as confounders, as the influence of these variables in arrhythmogenesis or sudden death has been previously suggested in horses or humans.^7^, ^40^, ^41^ The associations between the binary outcome of interest and independent variables of the model from the mixed‐effects linear regression model were reported as odds ratios (OR) with their respective 95% confidence intervals (95% CI). For Poisson regression models, associations between the outcome of interest and the independent variable of the model were reported as incidence rate ratios (IRR) with their respective 95% CI. Coefficients were reported for linear regression models. Fisher protected least significant difference was used to adjust for multiple comparisons. For the linear models, the model fits were assessed using visual inspection of the residuals against the predicted values. The pseudo *R* ^2^ and the Hosmer–Lemeshow goodness‐of‐fit procedure were used for the logistic regression models. Similarly, the Pseudo *R* ^2^ and post hoc goodness‐of‐fit were used to assess the fits of the Poisson regression models. For the linear models, the normality of the residuals was assessed using a quantal normal plot.

## 3 RESULTS

### 3.1 Description of arrhythmias and DFA‐α1 values in eventing horses during cross‐country competition

A convenience sample of 111 exercising ECGs from 54 horses obtained during cross‐country eventing competitions and corresponding competition results for each horse was available. After the elimination of unreadable ECG recordings, 69 exercising ECGs and competition results from 43 horses competing in eventing in two European countries between 2020 and 2022 were used for analysis. The duration (mean ± SD) of the exercising recordings was 5 min and 53 s ± 1 min and 9 s. Horses were competing in two different types of eventing competition: Fédération Équestre Internationale (FEI) sanctioned 2–4* Level competitions (FEI group) and competitions comprising FEI‐analogous competitive eventing training with cross‐country tests (non‐FEI group). The latter program took place as part of the COVID‐linked program by the Swiss Equestrian Federation in 2020 for the national eventing team and riders. Non‐FEI competitions resembled FEI Short format competitions; the details have been reported elsewhere.^42^ ECGs during FEI competitions were obtained as part of competition and training monitoring by the German equestrian federation. Thirty‐six ECGs were obtained from horses in the ‘FEI group’, and 33 were obtained from horses in the ‘non‐FEI group’. A total of 43 horses were recorded in (median, IQR [range]) 1, 1–2 [1–4] competitions. There were 23 mares and 20 geldings with an age of 12, 11–13 [7–19] years, and 45, 32–51 [16–100]% of Thoroughbred blood (in each horse). Fifteen recordings were made during 2* level competitions (9 2S[short] FEI, 6 2L[long] FEI), 29 during 3* level competitions (13 3S FEI, 6 3L FEI and 10 3 Non‐FEI) and 25 during 4* level competitions (2 FEI and 23 Non‐FEI). Recordings were obtained during 10 different events at 8 different venues throughout Europe.

The nPC/recording was median, IQR [Range] 2, 0–5 [0–70]. The HR_mean_ and HR_peak_ were (mean ± SD) 197 ± 9/min and 216 ± 11/min. There were 51 (74%) recordings from 35 horses (81%) with ARRHYTHMIA present. The types of arrhythmias are summarised in Table 1. There were 14 recordings with ARRHYTHMIA_COMPLEX_ present; 10 were during exercise and 4 were during recovery. The 14 recordings with ARRHYTHMIA_COMPLEX_ were from 11 different horses. Five of the 11 horses with ARRHYTHMIA_COMPLEX_ had one available recording. Three of the 11 horses with ARRHYTHMIA_COMPLEX_ had 2 available recordings each. Of the 3 horses with ARRHYTHMIA_COMPLEX_ and 2 available recordings, 1 horse showed ARRHYTHMIA_COMPLEX_ in both recordings and 2 horses in only one. For the remaining 3 horses with ARRHYTHMIA_COMPLEX_, 3 (*n* = 2) or 4 (*n* = 1) ECG recordings were available. One horse with three available recordings displayed ARRHYTHMIA_COMPLEX_ in all three recordings, and the other 2 horses displayed ARRHYTHMIA_COMPLEX_ in one recording. Detrended fluctuation analysis α1 was 0.64 ± 0.23 during the cross‐country phase of eventing competitions. Post‐exercise blood lactate concentration was 12.1 ± 5.8 mmol/L. Descriptive statistics of DFA‐α1, competition results, and markers of intensity of exercise according to arrhythmia categories are summarised in Table 2.

**TABLE 1:** Median IQR (range) of episodes of each rhythm disturbance category, number of recordings and number of horses with each rhythm disturbance category during cross‐country competition and the recovery period.

	Median, IQR (range) episodes per recording during exercise	Median, IQR (range) episodes per recording during recovery	Number of recordings with arrhythmia category during exercise/recovery	Number of horses with arrhythmia category during exercise/recovery
Single narrow complexes with a compensatory pause	1, 0–3 (0–49)	0,0 (0–19)	41/11	25/10
Single narrow complexes without a compensatory pause	0, 0 (0–6)	0	1/0	1/0
Single wide complexes with a compensatory pause	0	0, 0 (0–1)	0/2	0/2
Couplets narrow	0, 0 (0–6)	0, 0 (0–1)	15/1	10/1
Couplets wide	0, 0 (0–1)	0, 0 (0–1)	5/2	5/2
Triplets narrow	0, 0 (0–3)	0, 0 (0–1)	6/2	6/2
Triplets wide	0, 0 (0–1)	0	1/0	1/0
Salvos or runs narrow	0, 0 (0–2)	0	8/0	6/0
Salvo or runs wide	0	0, 0 (0–1)	0/1	0/1
Total	NA	NA	42/19	28/17

*Note*: One single, one couplet, one triplet and one salvo or run of premature complexes are considered one episode. Premature complexes (PCs) were described as wide or narrow based on subjective comparison with the appearance of the preceding QRS. NA, not applicable, Total number of recordings = 69. Total number of horses = 43. The duration (mean ± SD) of the exercising recordings was 5 min and 53 s ± 1 min and 9 s.

**TABLE 2:** Summary statistics of detrended fluctuation analysis α1 (DFA‐α1), competition results and markers of exercise intensity in horses with different arrhythmia categorisations.

	ARRHYTHMIA_COMPLEX_		ARRHYTHMIA_EXERCISE_		ARRHYTHMIA_RECOVERY_	
	YES; *n* = 14	NO; *n* = 45	YES; *n* = 42	NO; *n* = 27	YES; *n* = 19	NO; *n* = 50
DFA‐α1	0.75 ± 0.16	0.62 ± 0.24	0.70 ± 0.21	0.55 ± 0.23	0.62 ± 0.21	0.65 ± 0.24
HR_mean_ (beats/min)	199 ± 8	197 ± 9	198 ± 7	196 ± 32	198 ± 8	197 ± 9
HR_peak_ (beats/min)	221 ± 9	215 ± 11	218 ± 9	213 ± 35	217 ± 9	216 ± 12
Lactate (mmol/L)	12.3 ± 5.8	12 ± 9.4	12.5 ± 6.3	11.4 ± 5.1	11.7 ± 4.8	12.2 ± 6.3
Speed (100 m/min)	5.46, 5.31–5.9 [5.03–5.64]	5.44, 5.22–5.57 [3.92–6.34]	5.42, 5.22–5.52 [4.39–6.35]	5.46, 5.38–5.67 [3.91–6.30]	5.57, 5.44–5.86 [5.22–6.30]	5.41, 5.08–5.49 [3.91–6.35]
Distance (m)	32.17, 28.5–38.63 [27.5–46]	28.5, 28.5–34.15 [27.5–45.42]	30.32, 28.5–34.6 [27.5–46]	28.5, 28.5–32.9 [27.5–45.42]	28.5, 28.5–34.28 [27.5–46]	29.29, 28.5–43.5 [27.5–45.42]
Total penalties	39.8, 31.2–47.6 [25.2–105.2]	40.6, 31.5–51 [24.3–145.7]	40.4, 31.2–48.6 [24.9–145.7]	42.9, 34.9–56.3 [24.3–81.7]	**37.6, 31–53 [24.3–81.7]**	**42.3, 33–50.8 [24.9–145.7]**
Cross‐country time penalties	**1.6, 0–2.5 [0–17.2]**	**3.6, 0–7.6 [0–42.4]**	3.4, 0.7–7.8 [0–42.4]	1.6, 0–4.4 [0–21.2]	**0.8, 0–2.2 [0–6]**	**4.4, 0.6–9.6 [0–42.4]**
Showjumping penalties	**4.6, 1.3–8 [0–28]**	**4, 0–8 [0–38.4]**	4, 0–8 [0–28]	4, 0.9–8 [0–38.4]	**4, 1.3–8.9 [0–38.4]**	**4, 0–8 [0–28]**
Sex	10 g, 4 m	22 g, 33 m	21 g, 21 m	11 g, 16 m	8 g, 11 m	24 g, 26 m
Age (years)	13, 11.25–14 [11–19]	12, 11–13 [7–16]	12, 11–13 [7–19]	12, 10.5–13 [7–16]	12, 11–13 [9–19]	12, 11–13 [7–19]
% TH blood	43, 40–53 [23–72]	45, 31–51 [16–100]	43, 30.5–50 [16–76]	46, 41–54 [21–100]	52, 38–72 [27–100]	44, 31–50 [16–72]

*Note*: Results are described as median, IQR [range] or mean ± SD. Variables for which there is an association between rows and columns are in bold.

Abbreviations: g, gelding; m, mare; *N*, number of recordings.

Associations between competition results and exercising arrhythmias or DFA‐α1 are reported in Table 3. Associations between markers of exercise intensity (blood lactate concentration, HR_mean_, HR_peak_, speed and distance) and exercising arrhythmias, DFA‐α1 or competition results are reported in Table 4.

**TABLE 3:** Results of Poisson regression analysis with dependent variables listed in columns, independent variables listed in rows and confounders (age, sex and % of Thoroughbred blood) listed as superscripts for significant values.

	Dependent					
Independent	Total penalties		Cross‐country time penalties		Showjumping penalties	
	*p*	IRR (95% CI)	*p*	IRR (95% CI)	*p*	IRR (95% CI)
DFA‐α1	0.2	0.82 (0.63–1.077)	0.7	0.87 (0.39–1.95)	**0.03** ^a^	0.36 (0.15–0.88)
nPC	0.1	0.99 (0.99–1.0017)	**0.03**	0.97 (0.95–0.99)	0.8	1.002 (0.98–1.03)
ARRHYTHMIA_COMPLEX_	0.7	0.97 (0.83–1.14)	**<0.001**	0.27 (0.14–0.52)	**0.03** ^b^	1.69 (1.063–2.69)
ARRHYTHMIA_EXERCISE_	0.9	0.99 (0.87–1.12)	0.09	1.42 (0.94–2.15)	0.8	0.95 (0.65–1.39)
ARRHYTHMIA_RECOVERY_	**0.05** ^c^	0.87 (0.76–0.99)	**<0.001**	0.25 (0.14–0.45)	**0.003**	1.89 (1.25–2.87)

*Note*: The analysis evaluates the association between DFA‐α1, arrhythmias and competition results. Each row is a different multivariable analysis and confounders are described when significant and when associations of dependent and independent variables are significant. These are listed as superscripts a–c.

Abbreviations: CI, 95% confidence interval; IRR, incidence rate ratio; nPC, number of premature complexes; OR, odds ratio.

^a^ % of Thoroughbred blood was a modifier of this relationship *p* = 0.008; IRR = 23.78 (2.30–245.77).

^b^ % of Thoroughbred blood was a modifier of this relationship *p* = 0.01; IRR = 15.27 (1.901–122.376).

^c^ Sex, age and % of Thoroughbred blood were significant modifiers of this relationship *p* = 0.046, IRR = 0.87 (0.76–0.99); *p* = 0.005, IRR = 0.81 (0.65–0.996); *p* = 0.01, IRR = 2.195 (1.203–3.979).

**TABLE 4:** Results of linear, Poisson and logistic regression analysis evaluating the association between variables that describe DFA‐α1, arrhythmias and competition results set as dependent variables (columns) and markers of exercise intensity set as independent variables (rows).

	Dependent															
Independent	DFA‐α1		nPC		Arrhythmia complex		Arrhythmia exercise		Arrhythmia recovery		Total penalties		Cross‐country time penalties		Jumping penalties	
	*p*	Coefficient (95% CI)	*p*	IRR (95% CI)	*p*	OR (95% CI)	*p*	OR (95% CI)	*p*	OR (95% CI)	*p*	IRR (95% CI)	*p*	IRR (95% CI)	*p*	IRR (95% CI)
Lactate	0.6	−0.0031 (−0.013 to 0.0072)	**0.003** ^a^	1.041 (1.014–1.068)	0.8	1.026 (0.87–1.21)	0.5	1.03 (0.92–1.14)	0.6	1.03 (0.92–1.16)	**0.04** ^b^	0.98 (0.98–0.99)	**<0.001** ^c^	IRR = 0.90 (0.87–0.94)	0.3	0.98 (0.95–1.016)
HRmean	NA	NA	**<0.001** ^d^	1.074 (1.045–1.10)	0.6	1.029 (0.94–1.13)	0.6	1.014 (0.95–1.074)	0.3	1.038 (0.97–1.11)	0.3	0.99 (0.99–1.004)	**<0.001**	0.94 (0.91–0.7)	0.7	0.99 (0.94–1.019)
HRpeak	NA	NA	**<0.001** ^e^	1.047 (1.030–1.062)	0.2	1.063 (0.97–1.17)	0.2	1.03 (0.98–1.084)	0.3	1.03 (0.97–1.09)	**0.04** ^f^	0.99 (0.99–1)	**<0.001**	0.96 (0.94–0.98)	0.2	0.98 (0.97–1.005)
CC Speed	0.08	−0.11 (−0.24 to 0.013)	**0.005** ^g^	2.24 (1.27–3.93)	0.6	1.85 (0.18–18.50)	0.3	0.47 (0.12–1.73)	**0.02** ^h^	OR = 9.31 (1.48–58.45)	NA	NA	NA	NA	**0.006** ^i^	2.578 (1.32–5.043)
CC distance	0.4	−0.0043 (−0.015 to 0.0062)	**<0.001**	IRR = 1.080 (1.036–1.125)	0.2	1.23 (0.92–1.67)	0.7	1.02 (0.92–1.12)	0.9	0.99 (0.98–1.12)	NA	NA	NA	NA	0.4	0.98 (0.94–1.023)

*Note*: Each row is a different multivariable analysis. Age, sex and % of Thoroughbred blood were introduced as confounders, and results are listed for significant associations as superscripts a–i.Variables in which the dependent variable is a direct computation result of the independent variable or when the association is not part of the study objectives were not analysed and are labelled as NA.

Abbreviations: CI, 95% confidence interval; IRR, incidence rate ratio; nPC, number of premature complexes; OR, odds ratio.

^a^ Age was a modifier of this relationship *p* = 0.009, IRR = 1.29 (1.067–1.57).

^b^ Sex and age were modifiers of this relationship *p* = 0.02, IRR = 0.77 (0.62–0.95); *p* = 0.002, 0.92 (0.88–0.97).

^c^ Sex was a modifier of this relationship *p* = 0.04, IRR = 0.407 (0.17–0.95).

^d^ Age was a modifier of this relationship *p* = 0.009, IRR = 1.32 (1.072–1.62).

^e^ Age was a modifier of this relationship *p* = 0.04, IRR = 1.22 (1.004–1.48).

^f^ Sex and age were modifiers of this relationship *p* = 0.02, IRR = 0.79 (0.64–0.96); *p* = 0.004, 0.94 (0.90–0.98).

^g^ Age was a modifier of this relationship *p* = 0.04, IRR = 1.23 (1.013–1.45).

^h^ % of Thoroughbred blood was a modifier of this relationship *p* = 0.02, IRR = 211.51 (2.87–15 575.67).

^i^ % of Thoroughbred blood was a modifier of this relationship *p* = 0.045, IRR = 11.43 (1.052–124.2).

## 4 DISCUSSION

### 4.1 Description of arrhythmias and DFA‐α1 values in eventing horses during cross‐country competition

Arrhythmias were common in eventing horses competing without known signs of cardiovascular disease. This is consistent with reports of exercising rhythm in eventers and other equine athletes during training, exercise testing and competition as well as in fit human athletes.^43^ The frequency of arrhythmias in the current study (74% of recordings and 81% of horses displayed arrhythmias) approximates that reported in a recent report of cross‐country competition^7^ and is higher than that reported in standardised exercise tests in healthy eventers.^6^ Average and peak heart rates were higher than those reported for other sport horses during training.^3^, ^6^, ^44^ More frequent arrhythmias are often found as the intensity of exercise, estimated by heart rate, increases,^1^, ^6^, ^45^ and this could explain the more frequent presence of arrhythmias in the group of horses presented here. Circumstances surrounding exercise and familiarity with the test have also been suggested to affect arrhythmogenesis.^46^, ^47^ It is possible that the competition environment was another contributing factor to the higher frequency of arrhythmias in horses during competition reported here compared with studies that evaluate arrhythmias during training.^6^, ^48^ It is a limitation of the study that horses did not have complete cardiac evaluations and were included based on the absence of reported cardiovascular signs and reported good performance. For this reason, it cannot be ruled out that some of the horses in the study had underlying cardiovascular disease.

Detrended fluctuation analysis α1 was described during cross‐country competition (0.64 ± 0.23) in the horses in this study. In human athletes, DFA‐α1 values of 0.75 and 0.5 have been suggested to be associated with thresholds that mark the transition from the moderate to heavy exercise intensity domain and from the heavy to severe intensity domains, respectively. The threshold between the heavy and severe intensity domain is sometimes considered to approximate the anaerobic threshold, second ventilatory threshold, or second blood lactate threshold.^14^, ^18^, ^49^ Post‐exercise blood lactate concentration was 12.1 ± 5.8 mmol/L in the current study, suggesting that the intensity of exercise was, as expected, high. DFA‐α1 may have different patterns or absolute values in horses when compared with humans, or the average of DFA‐α1 during exercise with frequent accelerations, decelerations, inclines, or jumps does not reflect the complexity of the effort during eventing.^36^ It should also be noted that when using artefact correction algorithms for HRV analysis, eliminated RR values are replaced using interpolation and this affects results variability. In the current study, we elected to eliminate premature complexes using a restrictive criterion. A different methodology may be more appropriate for other purposes or to answer questions. Heart rate variability is associated with the presence of arrhythmias and with musculoskeletal disease^50^, ^51^ and has the potential to be useful for automatisation of arrhythmia detection or detection of subclinical disease.

### 4.2 Associations between competition results and exercising arrhythmias or DFA‐α1

More nPCs, ARRHYTHMIA_COMPLEX_ and ARRHYTHMIA_RECOVERY_ were associated with fewer time penalties during cross‐country. Arrhythmias during recovery were also associated with a lower number of total penalties. This finding supports previous information suggesting that many arrhythmias may be part of the physiological response to intense exercise. It is possible that arrhythmias are more frequent in horses that exercise at higher intensities and that a higher intensity or level of effort is associated with better results during competition. It is also possible that the training process that elicits the adaptations present in more successful athletes could elicit proarrhythmic changes, as it has been suggested that the athlete's heart is a proarrhythmic heart.^52^, ^53^ It should be noted that more severe arrhythmias than those detected in this study can cause performance and safety problems. Thresholds or categorisation of arrhythmias that should be considered relevant for performance or safety cannot be defined by the current or previous studies but have been suggested by a panel of experts in equine and human cardiology.^54^, ^55^

We included triplets as a complex arrhythmia and couplets as a noncomplex arrhythmia following recommendations for ‘Eligibility and Disqualification Recommendations for Competitive (human) Athletes With Cardiovascular Abnormalities’ written by the American Heart Association and American College of Cardiology in which ‘no greater than (ventricular) couplets’ are the complex forms ‘permitted’ in the absence of structural heart disease before exercise restriction or advance testing is recommended.^55^ Further sub‐classification of arrhythmias could be interesting and was used descriptively here and in a recently published article with a similar group of competitive eventers.^7^ For statistical analysis, limiting the number of categories was preferred to retain statistical power and answer the study questions. Interestingly, sex, age and percentage of Thoroughbred blood modified the relationship between the arrhythmias during recovery and the total number of penalties with geldings, older horses and horses with a higher percentage of Thoroughbred blood having more total penalties. The study was not specifically designed to study the effects of these variables on arrhythmogenesis, and the nature of the relationship cannot be elucidated with the current methodology. These variables may be relevant to arrhythmogenesis studies.

The presence of ARRHYTHMIA_COMPLEX_ and ARRHYTHMIA_RECOVERY_ was associated with more penalties during the showjumping phase. A higher DFA‐α1, suggested to represent a lower exercise intensity, was also associated with lower showjumping penalties. The rhythm during the showjumping phase was not recorded, and therefore, a direct negative effect of arrhythmias during showjumping performance was not evaluated. A plausible physiologic explanation is that the arrhythmias and lower DFA‐α1 during cross‐country are phenomena associated with higher exercise intensity, and this may contribute to higher penalties during showjumping, a test that occurs the day following cross‐country. Following the same speculative rationale, a higher speed during cross‐country was associated with more showjumping penalties in this study. Interestingly, for these two associations, the % of Thoroughbred blood was a modifier (a higher percentage associated with higher showjumping penalties). These should be interpreted cautiously as the associations are quantitatively small or have wide confidence intervals.

### 4.3 Associations between markers of exercise intensity (blood lactate concentration, HR_mean_ , HR_peak_ , speed and distance) and exercising arrhythmias, DFA‐α1 or results

The hypothesis that a lower DFA‐α1 would be associated with markers of exercise intensity was rejected. An association between DFA‐α1 and blood lactate has been previously described in horses and humans performing standardised exercise tests.^11^, ^12^, ^13^ The concept of using HRV as a non‐invasive tool to describe and monitor the intensity of effort is attractive. These previously reported associations are mechanistically interesting and one of the motivations of the study. However, the results do not demonstrate a clear benefit of monitoring DFA‐α1 in the context of eventing cross‐country competition as a proxy for exercise intensity or fatigue. The association of DFA‐α1 with showjumping penalties described above is interesting, but firm conclusions about its practical usefulness cannot be drawn.

The number of premature depolarisations used as an overall marker of arrhythmogenesis was positively associated with all the measured markers of exercise intensity. There was an association between ARRHYTHMIA_RECOVERY_ and the speed during cross‐country, and no associations with ARRHYTHMIA_COMPLEX_ or ARRHYTHMIA_EXERCISE_ were found. This is in keeping with previous literature that shows the frequent presence of more exercising arrhythmias in horses exercising at higher intensities.^1^, ^6^, ^45^ Age was a modifier of this relationship, with increasing age associated with developing more PCs, as previously shown.^7^

Higher blood lactate concentration, HR_peak_ and HR_mean_ were associated with fewer time penalties during cross‐country, and higher lactate and HR_peak_ with fewer total penalties. This may reflect that those horses capable or willing to perform at higher intensity have better competitive results. A higher blood lactate concentration after exercise has been previously associated with running speed in horses^30^, ^36^, ^42^ and a larger blood lactate production rate is thought to be advantageous in human sports that include high‐intensity exercise periods.^56^ A lower blood lactate concentration is sometimes used as a marker of superior fitness or readiness to perform in horses. This may be appropriate in submaximal standardised exercise tests but not after maximal or near‐maximal efforts such as cross‐country competition. A high blood lactate concentration may be interpreted as the consequence of activating glycolytic energy pathways that are relevant at high intensities and prevalent in ‘fast twitch’ muscle fibres.^28^, ^57^ It could be proposed that well‐developed glycolytic activity may be advantageous in exercise that includes bursts of high intensity^58^ and jumps. This idea is speculative, and the current methodology does not permit answering questions regarding the nature of the associations described between competition results, heart rate and blood lactate concentration. There are limitations to using HR_peak_ or HR_mean_ as markers of exercise intensity. One limitation is that the maximal heart rate of horses is variable, and it was not known for horses included in this study. Riders of sport horses are often averse to exercise intensities that elicit maximal heart rate due to a perceived high risk for injury. Using a percentage of maximal heart rate or other measurements, such as oxygen consumption, would have been an interesting but near‐impossible approach in a competition setting.

## 5 CONCLUSIONS

Arrhythmias were frequent during cross‐country competition in horses without a history of poor performance or cardiovascular disease. Detrended fluctuation analysis α1 was not associated with performance markers during cross‐country, and a higher value of DFA‐α1 was associated with fewer penalties during the show‐jumping phase. Arrhythmias during cross‐country competitions were associated with fewer penalties during cross‐country and more penalties during the jumping phase. The nature of the association between arrhythmias and performance cannot be elucidated with the current study. However, it is plausible that this is not a cause –effect relationship and that higher exercise intensity or adaptations to training are mechanisms involved in the relationship between performance and the presence of arrhythmias. Sex, age and the percentage of Thoroughbred blood modify the relationships between arrhythmias, performance and exercise intensity and should be considered when studying arrhythmogenesis in sport horses. The specific arrhythmia types and circumstances that should raise concerns about performance and safety remain unanswered and relevant questions for equine practitioners.

## FUNDING INFORMATION

This research did not receive any specific grant from funding agencies in the public, commercial, or not‐for‐profit sectors.

## CONFLICT OF INTEREST STATEMENT

The authors have declared no conflicting interests.

## AUTHOR CONTRIBUTIONS

**Cristobal Navas de Solis:** Conceptualization; methodology; writing – original draft; writing – review and editing; project administration; supervision; formal analysis. **Alessandra Ramseyer:** Conceptualization; methodology; formal analysis; data curation; writing – review and editing; resources. **Darko Stefanovski:** Conceptualization; data curation; formal analysis; writing – review and editing. **Joanne Haughan:** Conceptualization; methodology; writing – review and editing. **Claire J. Solomon:** Conceptualization; methodology; data curation; formal analysis; writing – review and editing. **Katharina Kirsch:** Conceptualization; methodology; data curation; formal analysis; visualization; writing – review and editing.

## DATA INTEGRITY STATEMENT

Cristobal Navas de Solis had full access to all the data in the study and takes responsibility for the integrity of the data and the accuracy of the data analysis.

## ETHICAL ANIMAL RESEARCH

Protocols for this prospective longitudinal cohort study were performed with the approval of the appropriate committees for animal use and experimentation (VD2809).

## INFORMED CONSENT

Owner's informed consent was obtained.

## ANTIMICROBIAL STEWARDSHIP POLICY

Not applicable.

## Data Availability

The data that support findings of this study are available from the corresponding author upon reasonable request: Open sharing exemption granted by the editor due to lack of provision in the owner informed consent process.
